# Arabica coffee leaf images dataset for coffee leaf disease detection and classification

**DOI:** 10.1016/j.dib.2021.107142

**Published:** 2021-05-16

**Authors:** Jennifer Jepkoech, David Muchangi Mugo, Benson K. Kenduiywo, Edna Chebet Too

**Affiliations:** a University of Embu, P.O BOX 6 – 60100, Embu, Kenya; b Jomo Kenyatta University of Science and Technology, P.O. Box 62 000 – 00200, Nairobi, Kenya; c Chuka University, P.O BOX 109-60400, Chuka, Kenya

**Keywords:** Arabica coffee, Image datasets, Machine learning, Deep learning, Disease diagnosis

## Abstract

This article introduces Arabica coffee leaf datasets known as JMuBEN and JMuBEN2. Image acquisition was done in Mutira coffee plantation in Kirinyaga county-Kenya under real-world conditions using a digital camera and with the help of a pathologist. JMuBEN dataset contains three compressed folders with images inside. The first file contains 7682 images of Cerscospora, the second contains 8337 images of rust and the last one contains 6572 images of Phoma. JMuBEN2 contains two compressed files where the first file contains 16,979 images of Miner while the other contains 18,985 images of healthy leaves. In total, the dataset contains 58,555 leaf images spread across five classes (Phoma, Cescospora, Rust, Healthy, Miner,) with annotations regarding the state of the leaves and the disease names. The Arabica datasets contain images that facilitates training and validation during the utilization of deep learning algorithms for coffee plant leaf disease recognition and classification. The dataset is publicly and freely available at https://data.mendeley.com/datasets/tgv3zb82nd/1 and https://data.mendeley.com/datasets/t2r6rszp5c/1 respectively.

**Specifications Table**

**Table:** 

Subject	Computer Science, Agricultural Science, Biological Science
Specific subject area	Image processing
Type of data	Raw data, images
How data were acquired	Data acquisition was done using a Fujifilm X-T4 camera with a sensor size of APS-C, resolution of 26.1MP,viewfinder of 3690 K dots, monitor of 3.0-inch tilt-angle touchscreen, 1620 K dots, autofocus of 425-point AF and maximum continuous shooting rate of15fps (mechanical shutter), 30fps (electronic). Data acquisition was done with the help of a pathologist and annotation was done manually with the help of data labelling web tool.
Data format	The data are in jpeg format
Parameters for data collection	Images were taken on sunny, windy and cloudy days. The images correspond to both back and upper sides of healthy and infected coffee leaves.
Description of data collection	Data was collected using a Fujifilm X-T4 camera and with the help of a pathologist.
Data source location	Source location was central Kenya and specifically at Mutira coffee plantation in Kirinyaga county with a Latitude 0° 28′ 59″ S and a longitude of 37° 19′ 59″ E.
Data accessibility	The datasets are publicly and freely available on mendeley data repository with doi:10.17632/t2r6rszp5c.1 at https://data.mendeley.com/datasets/t2r6rszp5c/1 and doi:10.17632/tgv3zb82nd.1 at https://data.mendeley.com/datasets/tgv3zb82nd/1

**Value of the Data**

• Data is used for evaluating algorithms, which are used in machine learning or deep learning for training, testing and validation of classification of diseases associated with coffee leaves for instance Miner, Phoma, Cescospora and Rust.
• Data encourages and motivates further research in to plant leaf disease detection especially in the area of machine learning.
• The images in the two datasets are annotated and ready to be used in machine learning. This data can be used in improving the accuracy of arabica coffee leaf disease detection and classification because the machine does not need to learn extra background features.

## 1 Data Description

Table 1 above shows the distribution of images in each of the compressed files in the two datasets. JMuBEN dataset contains three compressed files with images inside. The first file contains 7682 images of Cerscospora, the second contains 8337 images of rust and the last one contains 6572 images of Phoma. JMuBEN2 contains two compressed files where the first file contains 16,979 images of Miner while the other contains 18,985 images of healthy leaves. In total, the dataset contains 58,555 leaf images spread across five classes (Phoma, Cescospora and Rust Healthy, Miner,) with annotations regarding the state of the leaves and the disease names. Specific description of each disease is explained below;

**Table 1:** Conditions of the leaves with the number of images for each condition.

Condition of Leaf	Number of images
Healthy	18,985
Rust	8337
Miner	16,979
Phoma	7682
Rust	6572

### 1.1 Rust

**Fig. 1:**
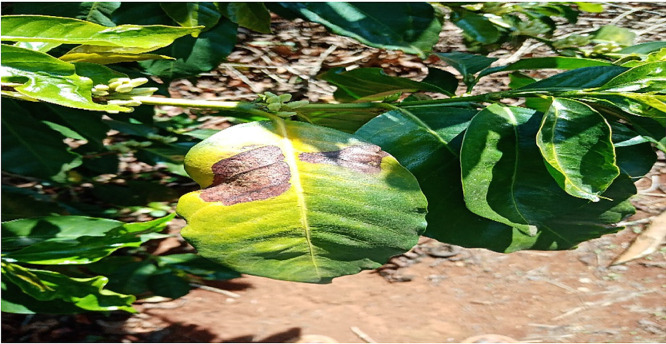
An image of a leaf affected by Coffee Leaf Rust.

The pathologist and the researchers took a tour around the coffee plantation; the pathologist identified coffee leaf rust by observing the leaves that small yellow-orange and powdery lesions. The pathologist checked the leaves that had chlorotic patches on the upper surface and rust pustules on the under leaf surface. Pictures of such leaves were taken and later label as Rust. The pathologist explained that rust is caused by hemileia vastatrix and is spread to the lower side of the leaf by wind and rainwater. After identification, pictures of the affected leaves were harvested using a digital calera. . A total of 8337 images were taken and processed in this class. Fig. 1 below shows the image of a leaf affected by Rust.

### 1.2 Phoma

**Fig. 2:**
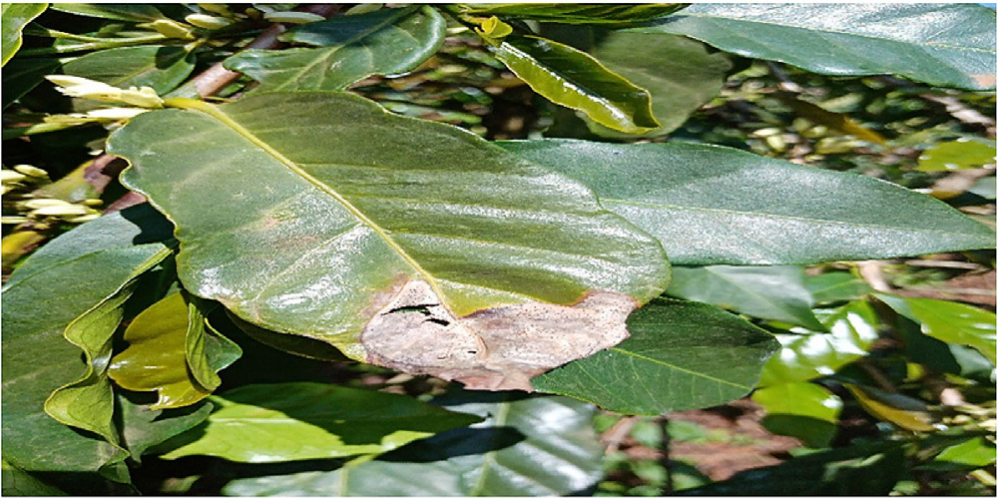
An image of a leaf affected by Phoma.

Researchers together with the plant pathologist moved around the farm and used observation method [2] to identify the leaves whose trees were beginning to die from the tip of the leaves. According to the pathologist, a leaf that dies from the tip area towards the other sides [4] is a strong indication that the leaf is suffering from phoma. Such leaves were identified and images taken using a digital camera for further processing. A total of 6572 images were taken and processed in this class. Fig. 2 below shows an image of leaf affected by Phoma.

### 1.3 Miner

**Fig. 3:**
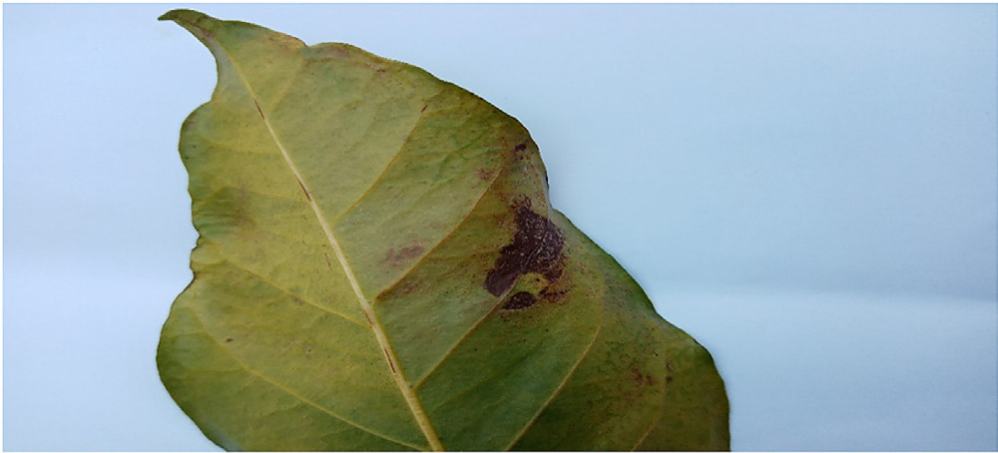
An image of a leaf affected by Miner.

During the feeding time of miner larvae according to the pathologist, yellow trails are left underneath the coffee leaf epidermis. With the description, the plant pathologist moved through the entire coffee plantation and used observation [3] to identify the affected leaves. The identified leaves were pictured using a digital camera and stored for further processing. 16,979 images were collected and processes for this class. Fig. 3 below shows an image of a leaf affected by miner.

### 1.4 Cescospora

**Fig. 4:**
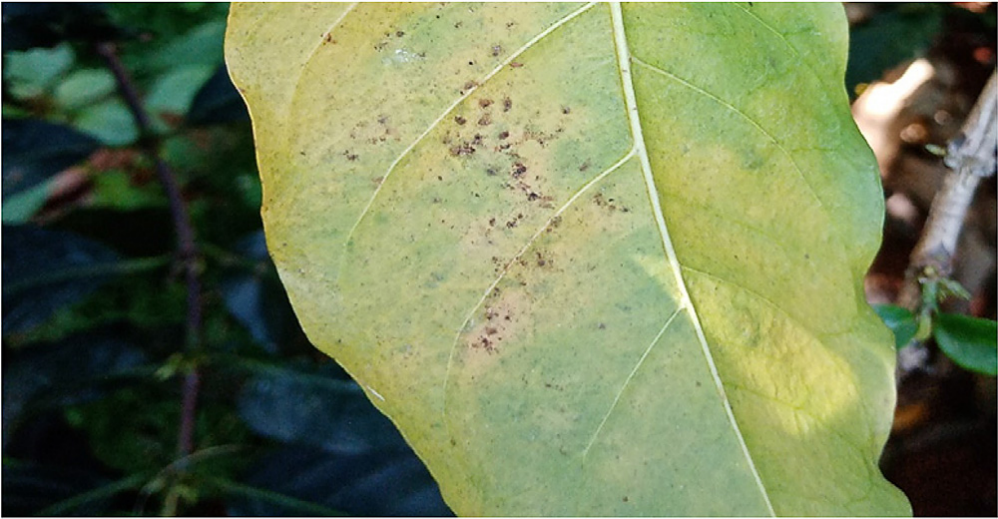
An image of a leaf affected by Cescospora.

Observation method [5] was used to identify this disease in coffee leaves. According to the pathologist, the appearance of circular grey,spots with tan, or white centers is a strong indication of the presence of Cescospora. Having this description, the researchers together with the pathologist took a tour of the entire coffee field identifying such leaves and taking the pictures using a digital camera following the method used by [6]. A total of 7682 were collected and processed for this class. Fig. 4 below shows an image of a leaf affected by miner.

## 2 Experimental Design, Materials and Methods

### 2.1 Data acquisition

This phase involved the acquisition of images from the field using a high-resolution digital camera and with the help of a pathologist who helped in disease description. A tour was taken through the coffee field by the researchers and the pathologist and as the pathologist described and identified the diseased leaves, the pictures were taken using the camera. In total, five image classes were collected which are Phoma, Rust and Cescospora, healthy and Miner.

## 3 Data pre-processing

### 3.1 Noise filtering

In the pre-processing phase, elimination of image misrepresentations and noises [1] was done to improve on image quality. The methods that were applied during data pre-processing include noise filtering and contrast stretching, which were done using mean and median filters. For mean filtering, the researchers used Gaussian and averaging filters. The averaging filters were used to remove grain noise from the images, where by local variations which were caused by grains reduced. Mean filtering was implemented as defined in [7]. The filters used masks over every pixel in the signal causing each of the constituents to be averaged together and form an exclusive pixel.

### 3.2 Cropping

**Fig. 5:**
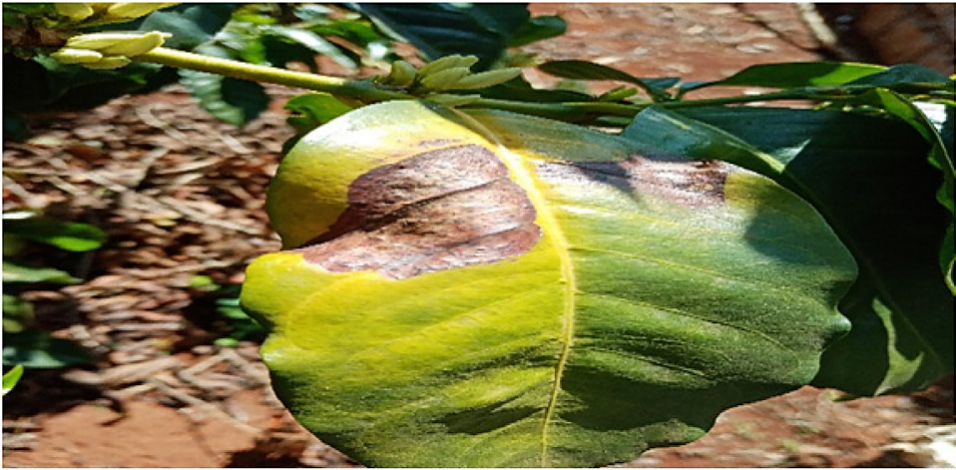
An image of a leaf affected by Coffee Leaf Rust before cropping.

**Fig. 6:**
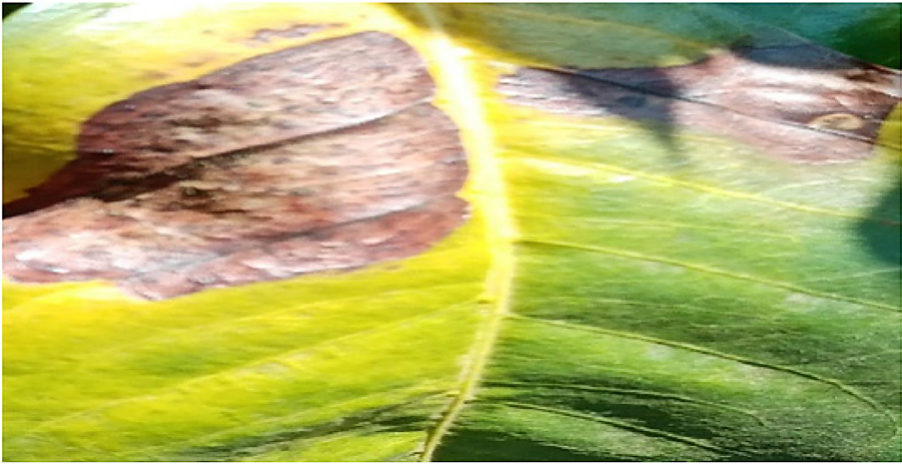
An image of a leaf affected by Coffee Leaf Rust after cropping.

Each image from the dataset was then checked to find out if they were of the same squared shape. The images that were not of the squared shape were cropped to get the centre square part of the image. Image dimensionality was also checked to make sure all images were equal in dimensionality. A crop tool was used to remove the unwanted areas of the images. Figs. 5 and 6 show an image before and after cropping. Cropping helps in emphasizing the area of interest in the leaf. This is an advantage to the deep learning researchers who may want to reduce training time in image processing.

### 3.3 Data augmentation

**Fig. 7:**
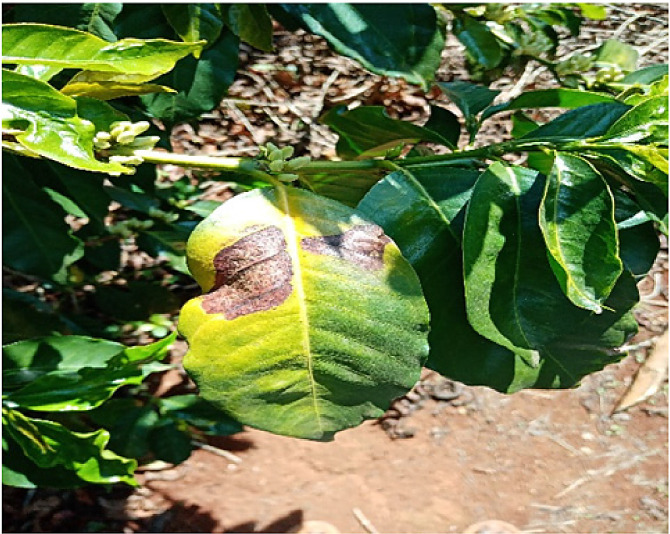
Image before rotation.

**Fig. 8:**
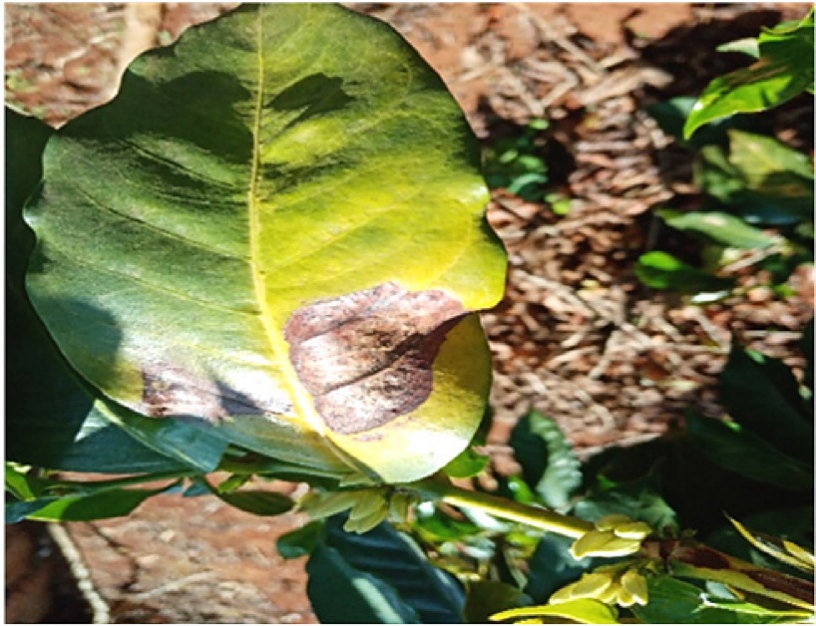
Image after rotation.

Data augmentation was done on the images that were collected from the field in order to improve smaller datasets by transforming them into large datasets. The data augmentation techniques that were used in this work include rotation and flipping. Rotation was done at an angle of 180° horizontally and vertically to emphasize the region of interest, which was affected by disease. In rotation, image rotator tool was used and was done counter clockwise. In the case of flipping, an image flipper was use. Some images were flipped horizontally using a horizontal flipper while others were flipped vertically using a vertical flipper. Flipping was done to enable better display of the diseased parts of the images. Figs. 7 and 8 below show an example image before and after rotation.

## Ethics Statement

We the authors assure consciously that for the article “**Arabica coffee leaf images dataset for coffee leaf disease detection and classification”** the following is fulfilled:

1) This article is the authors' own original work, which has not been previously published elsewhere.
2) The article is not currently being considered for publication elsewhere.
3) The article reflects the authors' own research and analysis in a truthful and complete manner.
4) The article properly credits the meaningful contributions of co-authors.
5) All authors have been personally and actively involved in substantial work leading to the article, and will take public responsibility for its content.

We agree with the above statements and declare that this submission follows the policies of Solid State Ionics as outlined in the Guide for Authors and in the Ethical Statement.

## CRediT Author Statement

**Jepkoech Jennifer:** Writing - review & editing; **Dr. David Mugo:** Writing - Original draft preparation; **Dr. Benson Kenduiywo:** Methodology, Conceptualization; **Dr. Edna Chebet:** Investigation of data collection methods.

## Declaration of Competing Interest

The authors of this work declare that they have no known competing financial interests or personal relation-ships that could have influenced the work reported here.
